# Chemerin/CMKLR1 Axis Promotes Inflammation and Pyroptosis by Activating NLRP3 Inflammasome in Diabetic Cardiomyopathy Rat

**DOI:** 10.3389/fphys.2020.00381

**Published:** 2020-04-23

**Authors:** Yebin Xie, Yu Huang, Xiaoyu Ling, Haiou Qin, Min Wang, Beibei Luo

**Affiliations:** ^1^Department of Geriatric Cardiology, The First Affiliated Hospital of Guangxi Medical University, Nanning, China; ^2^Guangxi Key Laboratory of Precision Medicine in Cardio-Cerebrovascular Diseases Control and Prevention, The First Affiliated Hospital of Guangxi Medical University, Nanning, China; ^3^Guangxi Clinical Research Center for Cardio-Cerebrovascular Diseases, The First Affiliated Hospital of Guangxi Medical University, Nanning, China

**Keywords:** chemerin, CMKLR1, NLRP3 inflammasome, pyroptosis, inflammation

## Abstract

Chemerin and its receptor CMKLR1 (a G-protein-coupled receptor) are inducers of inflammation, and play an important role in diabetic cardiomyopathy (DCM). In this study, we investigated the role of the chemerin/CMKLR1 axis in mediating inflammation and cell death in DCM. Sprague–Dawley rats, treated with a high-fat diet and low-dose of streptozotocin, were used as a DCM model. CMKLR1 expression was knocked down by siRNA (CMKLR1-siRNA) to evaluate the role of CMKLR1 in DCM. Chemerin-treated H9c2 cells were used to investigate the factors acting downstream of the chemerin/CMKLR1 axis. LDH release and EthD-III staining were used to measure the ratio of cell death *in vitro*. CMKLR1-siRNA and siRNA against nucleotide-binding oligomerization domain-like receptors 3 (NLRP3-siRNA) were used to explore the mechanism underlying chemerin-induced inflammation and cell death. The results showed that the expression of chemerin, CMKLR1, NLRP3, pro-caspase-1, activated caspase-1, and mature IL-1β was increased in the DCM model rat. Myocardium of DCM model rats exhibited fibrosis, hypertrophy, a disorganized ultrastructure, and impaired function. Pyroptosis was observed *in vivo* and *in vitro*. Silencing of CMKLR1 *in vivo* attenuated the expression of NLRP3 and activated caspase-1 and IL-1β. CMKLR1-siRNA treatment attenuated cardiac inflammation, fibrosis, hypertrophy, and pyroptosis, and improved cardiac function *in vivo*. Silencing of either CMKLR1 or NLRP3 suppressed the levels of activated caspase-1, IL-1β, and pyroptosis; however, silencing of both CMKLR1 and NLRP3 further decreased the levels of mature IL-1β and pyroptosis. Overall, the results showed that the chemerin/CMKLR1 axis contributed to the development of DCM and that the NLRP3 inflammasome mediated the chemerin/CMLR1-induced inflammation and pyroptosis. These data indicate that silencing of the *CMKLR1* gene might exert a protective effect against DCM.

## Introduction

The number of patients with diabetes worldwide is projected to reach approximately 642 million by 2040 (Ogurtsova et al., 2017). Cardiovascular disease is the most common complication of diabetes, and often leads to heart failure (Yap et al., 2019). Diabetic cardiomyopathy (DCM), characterized by disordered myocardial structure and function, is an important cause of heart failure among diabetic patients (Marwick et al., 2018). Previous research has indicated that inflammation is a critical inducer of cardiac remodeling, including cell death, fibrosis, and hypertrophy, which results in heart failure (Wang et al., 2016; Jin et al., 2017; Deckx et al., 2018; Wu et al., 2019; Yang L. et al., 2019). However, the mechanisms underlying the inflammatory processes in DCM remain unknown.

Chemerin, also known as tazarotene-induced gene 2 protein or retinoic acid receptor responder protein 2, is secreted by various cell types, including adipocytes, epithelial and endothelial cells, and fibroblasts (Helfer and Wu, 2018). Chemerin is secreted as an inactive precursor, pro-chemerin, which can be converted into the active form of chemerin following protease cleavage of the C-terminal domain. The main function of chemerin was initially described as the regulator of glucolipid metabolic processes and inflammatory responses (Helfer and Wu, 2018). Recently, however, chemerin was reported to be a key regulator of cardiovascular disease, including hypertension, coronary atherosclerosis disease, and myocardial infarction (Kadoglou et al., 2015). In patients with type 2 diabetes, elevated serum levels of chemerin have been associated with heart failure (Zhou et al., 2019) while chemerin was shown to induce murine cardiomyocyte apoptosis through inflammatory processes (Rodriguez-Penas et al., 2015). These results suggest that chemerin plays an important role in DCM by inducing cardiac inflammation.

Membrane-localized receptors for chemerin include G-protein-coupled chemokine-like receptor 1 (CMKLR1), G protein-coupled receptor 1 (GPR1), and chemokine (CC motif) receptor-like 2 (CCRL2) (Kaur et al., 2018). However, only CMKLR1 appears to be critical for chemerin-induced inflammation (Mariani and Roncucci, 2015). Chemerin binding to GPR1 produces only a weak effect in modulating calcium mobilization, while CCRL2 does not activate downstream signaling cascades (Stojek, 2017). In contrast, CMKLR1 has been reported to mediate chemerin-promoted inflammation in diabetic complication (Neves et al., 2018a, b; Zou et al., 2019). However, the role of the chemerin/CMKLR1 axis in DCM, as well as the associated underlying molecular mechanisms, remain unclear.

The NACHT, LRR, and PYD domains-containing protein 3 (NLRP3) inflammasome has been reported to regulate inflammatory processes in DCM. The NLRP3 inflammasome consists of NLRP3, apoptosis-associated speck-like protein containing a CARD (ASC), and the serine protease caspase-1 (Zhang et al., 2018; Kelley et al., 2019; Yang F. et al., 2019). Once activated, NLRP3 interacts with ASC, which induces the cleavage of pro-caspase-1 and formation of an active caspase-1 tetramer composed of two p20 and two p10 subunits (Kelley et al., 2019). The caspase-1 tetramer then cleaves pro-IL-1β, converting it into IL-1β, a critical step in myocardial apoptosis, hypertrophy, and fibrosis (Wang et al., 2016; Jin et al., 2017; Deckx et al., 2018; Zhang et al., 2018; Yang L. et al., 2019).

Activated caspase-1 can induce an inflammatory form of programmed cell death termed “pyroptosis” (Lacey et al., 2018), which shows characteristics typical of apoptosis and necrosis. Similar to apoptosis, cells undergoing pyroptosis incur DNA damage and become TUNEL-positive; and as with necrosis, pyroptosis exhibits membrane pore formation, release of proinflammatory cytoplasmic content, and cell rupture (Liu et al., 2016). EthD-III, a type of cell membrane-impermeant nucleic acid dye, stains pyroptotic cells by entering through the membrane pores, but does not stain cells with intact membranes (Pan et al., 2018; Wang X. et al., 2019). The permeability of pyroptotic cell membranes can also be determined through the release of the cytoplasmic enzyme lactose dehydrogenase (LDH) (Ding et al., 2019; Gu et al., 2019). Recent studies showed that pyroptosis also occurs in cardiomyocytes (Pan et al., 2018; Meng et al., 2019; Wang X. et al., 2019). In our previous study, we showed that NLRP3 inflammasome-induced caspase-1 activation promotes cardiomyocyte pyroptosis in DCM. However, the regulatory mechanism underlying NLRP3 inflammasome activation in DCM remains unknown.

The expression of chemerin and NLRP3 was shown to be upregulated in lung tissue following Limb I/R, possibly due to early responses to inflammation (Zou et al., 2019). Additionally, the chemerin/CMKLR1 axis was shown to induce the formation of the NLRP3 inflammasome as well as the expression of several proinflammatory cytokines in Kupffer cells (Zhang et al., 2017). However, little is known about the role of the chemerin/CMKLR1 axis in the regulation of the NLRP3 inflammasome in DCM.

The aim of the present study was to determine the role of the chemerin/CMKLR1 axis in DCM rats, and whether the NLRP3 inflammasome is an important mediator of the chemerin/CMKLR1 signaling-induced inflammatory effects in DCM.

## Materials and Methods

### Animals

Forty Sprague–Dawley rats (100–120 g) were divided into four groups (*n* = 10 per group) as follows: a control group (Ctrl), diabetic cardiomyopathy (DCM) group, DCM + negative control lentivirus (DCM+NC) group, and DCM+CMKLR1-siRNA group. All the rats were maintained at 22°C with 12 h light-dark cycles. The control group was fed a basal diet while the other groups were fed a high-fat diet (16% fat and 0.25% cholesterol). After 4 weeks, all groups underwent an intraperitoneal glucose tolerance test (IPGTT) and intraperitoneal insulin tolerance test (IPITT) to identify insulin resistance. To induce diabetes, rats in the DCM, DCM+NC, and DCM+CMKLR1-siRNA groups were administered a single intraperitoneal streptozotocin injection (STZ, 35 mg/kg; Solarbio, Beijing, China). Blood glucose levels were analyzed by blood glucose meter 1 week after STZ treatment (Roche, Switzerland). Rats with fasting blood glucose levels ≥11.1 mmol/L were considered diabetic. CMKLR1-siRNA treatment was carried out 8 weeks after STZ injection as this is when cardiac dysfunction occurs in diabetic rats (Lacombe et al., 2007; Ti et al., 2011). Rats from the DCM+NC and DCM+CMKLR1-siRNA groups were administered NC lentivector or CMKLR1-siRNA at a total dose of 1 × 10^8^ TU/rat (Genechem, China) by jugular vein injection. The sequences of the siRNAs used in this study were CMKLR1-siRNA: 5′-CAGUGAACAUGGUCUGGUU[dT][dT]-3′ (antisense); and NC: 5′-GCGCCAGUGGUACUUAAUATT-3′ (antisense). All the rats were euthanized under deep anesthesia 8 weeks after lentivector injection. Hearts were excised and immediately frozen to determine the transfection efficiency using fluorescence microscopy. The study protocol was approved by the Institutional Animal Care and Use Committee of the Guangxi Medical University. The study was performed in accordance with the National Institutes of Health (NIH) Guidelines for the Care and Use of Laboratory Animals.

### Echocardiography

Sixteen weeks after STZ injection, the rats were anesthetized with 10% chloral hydrate and transthoracic echocardiography was performed using the Vevo 770 imaging system with a RMB710 transducer (VisualSonics, Canada). The echocardiography parameters analyzed included left ventricular end-diastolic dimension (LVEDd), left ventricular ejection fraction (LVEF), fractional shortening (FS), and peak E to peak A ratio (E/A).

### Blood Pressure Assessment

Sixteen weeks after STZ injection, systolic blood pressure (SBP) and diastolic blood pressure (DBP) were evaluated in conscious rats using a tail-cuff system (Kent Scientific, United States).

### Histological Examination

The hearts were arrested with 10% KCl, and images of whole hearts were captured using a Canon camera (Tokyo, Japan). Sections (4 μm) were stained with hematoxylin and eosin (H&E). Cross section measurements were performed at the level of the nucleus in longitudinally sectioned myocytes. Images were obtained under a microscope (Olympus, Japan). Data were analyzed by Image-Pro Plus 6.0 (Media Cybemetics, United States).

### Immunohistochemistry

For immunohistochemistry, 4-μm tissue sections were incubated with primary antibodies against chemerin (1:100; Abcam, United Kingdom) and CMKLR1 (1:200; Abcam, United Kingdom) at 4°C overnight. Then, the sections were washed with phosphate-buffered saline (PBS) and incubated in secondary antibody for 30 min at 37°C, developed with daminobenzidine (DAB), and counterstained with hematoxylin. The images were obtained under a microscope (Olympus, Japan). Data were analyzed by Image-Pro Plus 6.0 (Media Cybemetics, United States).

### Masson’s Trichrome Staining

Masson’s trichrome staining was performed to determine the levels of collagen deposition in the hearts. Paraffin sections were stained using a Masson’s Trichorme Stain Kit (Solarbio, China). Images were obtained under a microscope (Olympus, Japan). Fibrotic areas were analyzed by Image-Pro Plus (Media Cybemetics, United States).

### TUNEL Assay

Paraffin sections were treated with xylene, rehydrated through a graded alcohol series, and then placed in 3% hydrogen peroxide in methanol for 10 min at room temperature. After adding the equilibration buffer, sections were treated with TdT-enzyme for 1 h at 37°C. The samples were then incubated with digoxigenin-conjugated antibodies for 30 min at 37°C. DAB was used as the staining agent. All procedures were performed following the manufacturer’s instructions (Millipore, United States).

For the TUNEL assay, cells were cultured on chamber slides, fixed in 4% paraformaldehyde for 30 min, and then permeabilized with Immunol Staining Wash Buffer (Beyotime, China) for 2 min on ice. The subsequent steps were as described in the section of TUNEL assay of paraffin samples. Images were captured under a microscope (Olympus, Japan). The TUNEL-positive ratio was assessed by Image-Pro Plus (Media Cybemetics, United States).

### Transmission Electron Microscopy

Approximately 1 mm^3^ of myocardium was obtained from the left ventricle and fixed in 2.5% glutaraldehyde in 0.1 mol/L sodium cacodylate buffer (pH 7.4) for 120 min, postfixed in buffered osmic acid, dehydrated in different alcohol concentrations, and embedded in Epon 812 mixture. Tissue sections (2 μm) were rinsed overnight in 0.1 mol/L phosphate buffer, postfixed for 2 h in 1% osmium tetroxide, dehydrated, and then embedded in an Araldite mixture. The sections were stained with uranyl acetate and lead citrate. Finally, images were obtained by transmission electron microscopy (TEM) (H-7650, HITACHI, Japan).

### Quantitative Real-Time RT-PCR

Total RNA was extracted from the left ventricle using TRIzol reagent (Invitrogen, CA, United States). Extracted RNA was reverse transcribed using a RevertAid First Strand cDNA Synthesis Kit (Thermo Fisher Scientific, United States). qPCR was performed using the Fast SYBR Green Master Mix (Thermo Fisher Scientific). The following primers were used: Chemerin forward, 5′-AAGGACTGGAAAAAGCCAGAG-3′ and reverse, 5′-TCCGGCCTAGAACTTTACCC-3′; CMKLR1 forward, 5′-GACCGGATTAGAACCCCAGT-3′ and reverse, 5′-AAAACCCCAAACCCATTAGC-3′; and β-actin forward, 5′-AGACCTTCAACACCCCAG-3′ and reverse, 5′-CACGATTTCCCTCTCAGC-3′. Relative mRNA expression was calculated by the 2^–△△CT^ method.

### Western Blot

Total protein was extracted from left ventricular or cell lysates using RIPA buffer and the content was measured using the Bicinchoninic acid assay (Beyotime). Protein samples were separated by 10–12% SDS-PAGE, transferred to nitrocellulose membranes (Millipore), and incubated with primary antibody. The following primary antibodies were used: anti-chemerin (1:700), anti-CMKLR1 (1:500), NLRP3 (1:500), caspase-1 (1:500), IL-1β (1:1,000) (all from Abcam), and β-actin (1:1,000, Santa Cruz Biotechnology, United States). Bands were revealed using a chemiluminescence reagent kit (Millipore) and quantified by densitometric analysis (Quantity One, Bio-Rad, United States).

### Enzyme-Linked Immunosorbent Assay

Fasting blood samples were collected from all groups after overnight fasting. Serum was obtained by centrifugation at 500 × *g* for 10 min and then stored at −80°C until analysis. The rat insulin ELISA kit was purchased from Jiancheng, Nanjing (China). The murine chemerin ELISA kit was purchased from R&D Systems (United Kingdom). The serum levels of insulin and chemerin were determined according to the manufacturer’s instructions.

### Cell Culture and Treatment

H9c2 cells (American Type Culture Collection) were cultured in normal glucose (5.5 mM) Dulbecco’s modified Eagle’s medium (DMEM) supplemented with 10% fetal bovine serum (FBS) at 37°C and 5% CO_2_. During the treatment period, and before stimulation, H9c2 cells were synchronized by serum starvation for 12 h. Chemerin was added to the medium at a concentration of 0–300 ng/mL for 0–48 h for evaluation of lactate dehydrogenase (LDH) release.

### Lentivirus Transfection

NC lentivirus, lentivirus-CMKLR1-siRNA, or lentivirus-CMKLR1-siRNA + lentivirus-NLRP3-siRNA were transfected into H9c2 cells at a multiplicity of infection (MOI) of 10. The sequence of the siRNA for rat NLRP3 was 5′-CCUGUCUUUGCCGTAGAUUACCGUAAG-3′ (antisense). Transfection efficiency was assessed by fluorescence microscopy. Cells were used only if CMKLR1 and/or NLRP3 mRNA levels were decreased by at least 70% compared with the control. Three parallel experiments were performed.

### LDH Release and EthD-III Staining

Supernatants from the treated cells were collected and centrifuged to remove solid particles. Lactate, INT, and diaphorase were mixed to produce the reaction mixture (Beyotime). Supernatants from each group were transferred into new 96-well plates and mixed with the reaction mixture for 30 min at room temperature. Serum-free medium was used as the 0% control and lysate of untreated cells was used as the 100% positive control. The absorbance value was analyzed at 490 nm. LDH release from each group was determined based on a standard curve.

EthD-III (Viability/Cytotoxicity Assay Kit, Biotium, United States) can enter into dead cells through membrane pores, producing an intense red fluorescence. Briefly, cells were incubated with 2 μM EthD-III at 37°C for 45 min and then stained with DAPI. Cells were visualized at 522/593 nm by confocal microscopy (LSM710, Carl Zeiss, Germany).

### Statistical Analysis

SPSS v18.0 (SPSS, Chicago, IL, United States) was used for data analysis. Data are shown as means ± SEM. Differences among experimental groups were analyzed by ANOVA, followed by the Tukey–Kramer post hoc test and independent samples *t*-test. *p* < 0.05 was considered significant.

## Results

### Diabetes Increased Chemerin and CMKLR1 Expression, and Knockdown of CMKLR1 by CMKLR1-siRNA in DCM Rats

**FIGURE 1:**
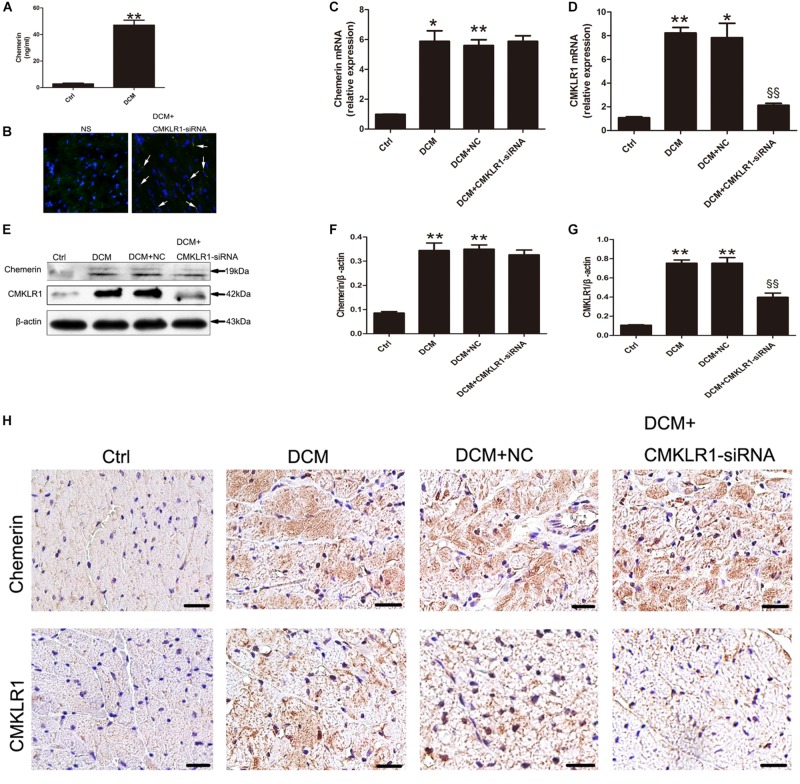
The expression of chemerin and CMKLR1 in diabetic cardiomyopathy (DCM) rats. **(A)** The serum levels of chemerin in the control and DCM groups. **(B)** Transfection efficiency of CMKLR1-siRNA in myocardium. Bright green points (white arrow) indicate GFP with lentivirus-CMKLR1-siRNA (scale bar: 20 μm). Relative mRNA **(C–D)** and protein **(E–G)** expression of chemerin and CMKLR1 in cardiac tissue with CMKLR1-siRNA treatment. Immunohistochemical staining **(H)** of chemerin and CMKLR1 in the different groups; scale bar: 20 μm. Data were presented as means ± SEM, *n* = 4–6. *p < 0.05, **p < 0.01 vs. Ctrl; ^§§^ *p* < 0.01 vs. DCM+NC. NC, negative control.

When compared with control rats, DCM rats showed increased serum levels of chemerin (Figure 1A; *p* < 0.01). The mRNA and protein levels of chemerin and CMKLR1 in left ventricle was increased in DCM rats than those in control (Figures 1C–G; *p* < 0.05∼*p* < 0.01). Four weeks after CMKLR1-siRNA treatment, transfection efficiency was evaluated in DCM rats received normal saline injection and DCM rat received CMKLR1-siRNA-injection. In the CMKLR1-siRNA group, more than 50% of myocytes were successfully transfected (Figure 1B). Western blot analysis revealed that CMKLR1-siRNA inhibited CMKLR1 upregulation in DCM rats (Figures 1E,G; *p* < 0.01), whereas it had no effect on chemerin (Figures 1E,F). Similar results were obtained with immunohistochemical staining (Figure 1H). In addition, immunohistochemistry clearly indicated the cytosolic expression of chemerin and membrane-localized expression of CMKLR1 in DCM rats when compared with control rats (Figure 1H).

### CMKLR1 Gene Silencing Inhibited NLRP3 Inflammasome Activation in DCM Rats

**FIGURE 2:**
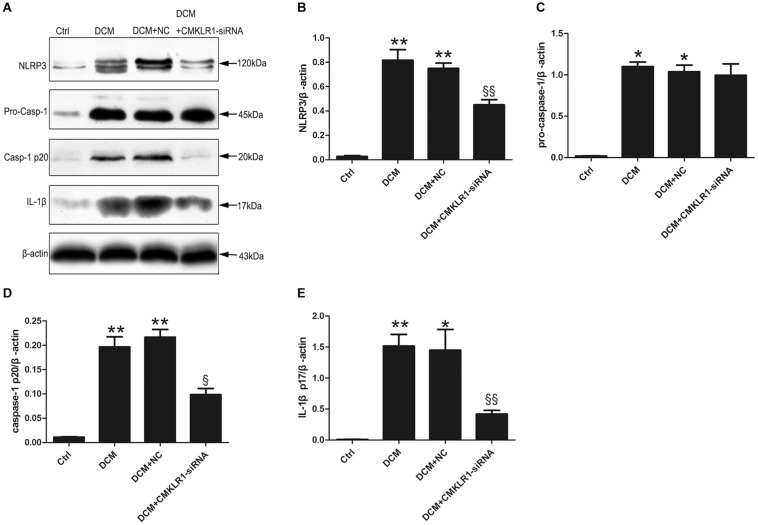
*CMKLR1* gene silencing inhibited the activation of the NLRP3 inflammasome in a rat model of diabetic cardiomyopathy (DCM). **(A–E)** Relative protein levels of NLRP3, pro-caspase-1, activated caspase-1, and mature IL-1β in the left ventricle of rats in the different groups. Data were presented as means ± SEM from three independent experiments. *p < 0.05, **p < 0.01 vs. Ctrl; ^§^ *p* < 0.05, ^§§^ *p* < 0.01 vs. DCM+NC. NC, negative control.

We then analyzed the protein expression of NLRP3, pro-caspase-1, activated caspase-1, and activated IL-1β in the rat myocardium, and found that the expression levels of all these proteins were higher in DCM rats than in control rats (Figure 2; *p* < 0.05∼*p* < 0.01). CMKLR1-siRNA suppressed the DCM-induced increase in the protein levels of NLRP3, activated caspase-1, and mature IL-1β, but not that of pro-caspase-1 (Figure 2; *p* < 0.05∼*p* < 0.01).

### CMKLR1 Gene Silencing Reversed the DCM-Induced Myocardial Remodeling

**FIGURE 3:**
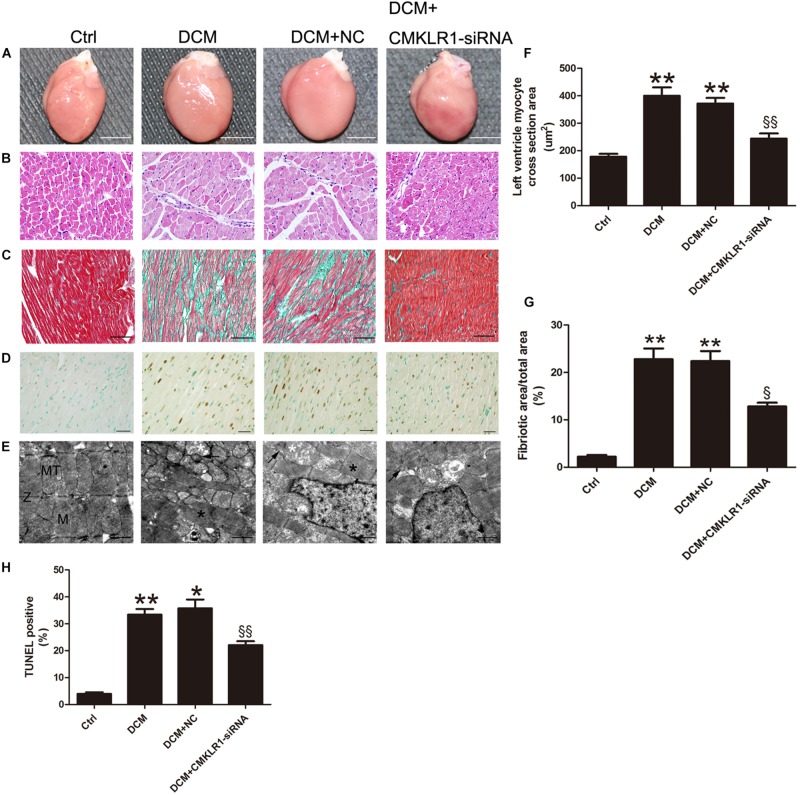
*CMKLR1* gene silencing reversed myocardial remodeling in diabetic cardiomyopathy (DCM) rats. **(A)** Heart size in the different groups; scale bar: 5 mm. **(B)** Hematoxylin and eosin (H&E) staining; scale bar: 20 μm. **(C)** Masson’s staining: fibrotic areas are stained green and normal cardiac myocytes are stained red; scale bar: 50 μm. **(D)** TUNEL staining of myocardium: dark brown staining indicates fragmented nuclear DNA; scale bar: 20 μm. **(E)** Transmission electron microscopy of rat hearts; M, normal myofibrils; MT, normal mitochondria; Z, normal Z-lines; black asterisk: disorganized myofibrils; white asterisk: swollen mitochondria; black arrow: accumulated lipids; scale bar: 1 μm. **(F)** Quantitative analysis of the cross-section area in H&E staining. **(G)** Quantitative analysis of the ratio of fibrotic area to total area. **(H)** Quantitative analysis of TUNEL-positive cells in each group. Data were presented as means ± SEM, *n* = 4–6. * *p* < 0.05 vs. Ctrl; ** *p* < 0.01 vs. Ctrl; ^§^ *p* < 0.05 vs. DCM+NC; ^§§^ *p* < 0.01 vs. DCM+NC.. NC, negative control.

Cardiac hypertrophy was observed in DCM rats (Figures 3A,B). The cross-section area of left ventricle cardiomyocyte from DCM rats was increased compared with the control group (Figure 3F; *p* < 0.01). TUNEL staining results showed that the ratio of dead cells in DCM group was higher than that of the control group (Figures 3D,H; *p* < 0.01). The level of interstitial cardiac fibrosis was markedly higher in the DCM group than in the controls (Figures 3C,G; *p* < 0.01). Symmetric myofibrils, well-organized Z lines with sarcomeres, and packed mitochondria beside the fibers could be observed in the cardiomyocytes of control rats (Figure 3E). In contrast, DCM rats exhibited destruction of myofibrils, swollen mitochondria with disorganized cristae, and lipid accumulated (Figure 3E).

With *CMKLR1* gene silencing, the myocardiocyte cross-section area, cardiac fibrosis area, and TUNEL-positive ratio were reduced in the DCM+CMKLR1-siRNA group compared with the NC group (Figures 3B–D,F–H; *p* < 0.05∼*p* < 0.01). CMKLR1-siRNA treatment reversed the changes in myofibrils, mitochondria, and lipid accumulation in the diabetic rats (Figure 3E).

### CMKLR1 Gene Silencing Improved Cardiac Dysfunction in DCM Rats

**FIGURE 4:**
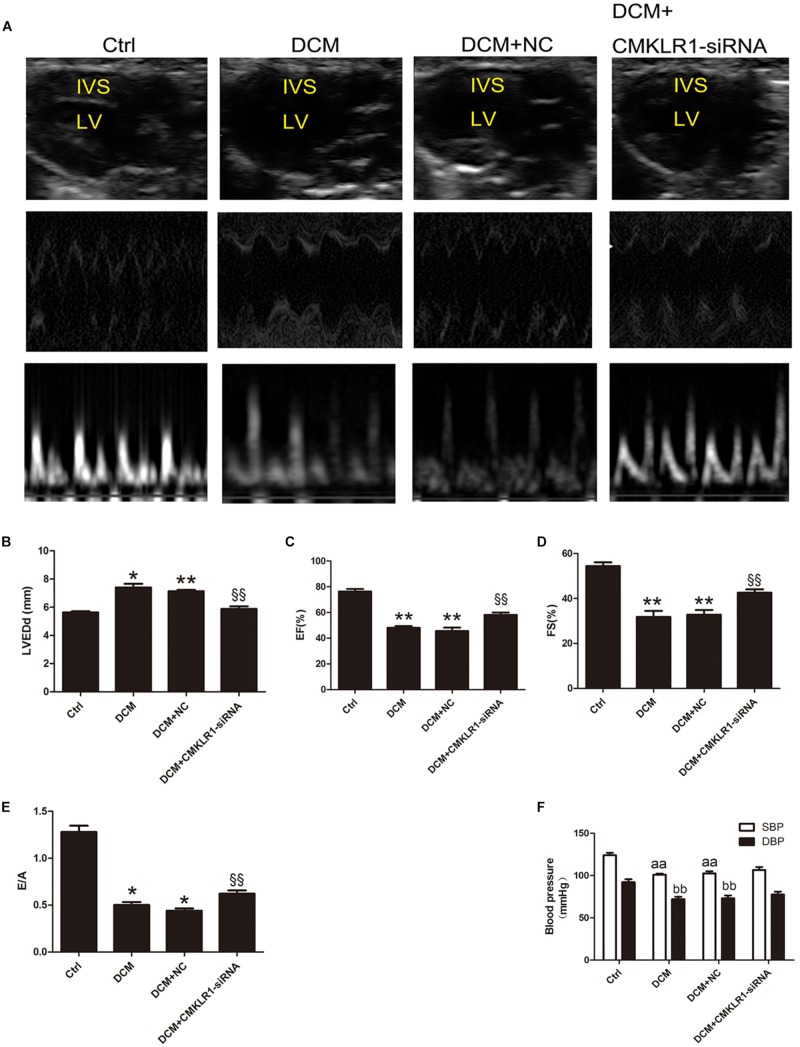
*CMKLR1* gene silencing improved cardiac dysfunction in diabetic cardiomyopathy (DCM) rats. **(A)** The cardiac function of rats in the different groups shown by echocardiography. Representative images of 2D echocardiograms, M-mode echocardiograms, and pulse-wave Doppler echocardiograms of mitral inflow. **(B–E)** Evaluation of LVEDd, EF, FS, and E/A. **(F)** SBP and DBP of each group at the end of study. Data were presented as means ± SEM, *n* = 6. *p < 0.05, **p < 0.01 vs. Ctrl; ^§§^ *p* < 0.01 vs. DCM+NC, ^aa^*p* < 0.01 vs. SBP of Ctrl, ^bb^*p* < 0.01 vs. DBP of Ctrl; NC, negative control; LV, left ventricle; IVS, interventricular septum; LVEDd, left ventricular end-diastolic dimension; EF, left ventricular ejection fraction; FS, fractional shortening; E/A, peak E to peak A ratio; SBP, systolic blood pressure; DBP, diastolic blood pressure.

Echocardiography showed that LVEDd was larger in DCM rats than in controls (Figures 4A,B; *p* < 0.05). EF, FS, and E/A were lower in DCM rats than in controls (Figures 4A,C–E; *p* < 0.05∼*p* < 0.01). Blood pressure analysis showed that SBP and DBP were both lower in DCM rats than in controls (Figure 4F; *p* < 0.01). Compared with the DCM+NC group, DCM+CMKLR1-siRNA group exhibited increased EF, FS, and E/A, and decreased LVEDd (Figures 4A–E; *p* < 0.05∼*p* < 0.01); however, no differences were observed in SBP or DBP between the two groups (Figure 4F).

### Chemerin Induced LDH Release in H9c2 Cardiomyocytes

**FIGURE 5:**
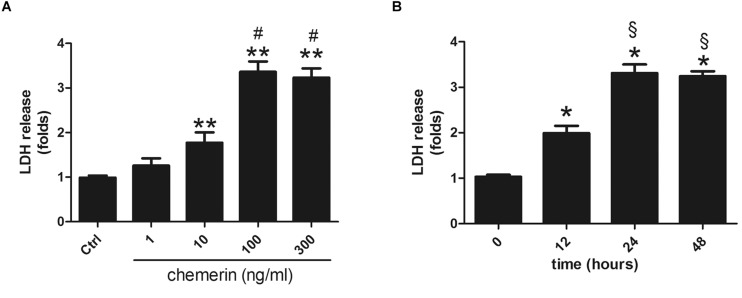
Chemerin induced LDH release in H9c2 cardiomyocytes. **(A)** Relative secreted level of LDH in H9c2 cells treated with different doses of chemerin. **(B)** The relative level of LDH in 100 ng/mL chemerin-treated H9c2 cells for different lengths of time. Data were presented as means ± SEM from three independent experiments. *p < 0.05, **p < 0.01 vs. Ctrl; ^ #^*p* < 0.05 vs. 10 ng/mL chemerin; ^§^ *p* < 0.05 vs. chemerin treatment for 12 h.

H9c2 cardiomyocytes were incubated with different concentrations of chemerin (0, 1, 10, 100, or 300 ng/mL) for 24 h. LDH release was higher in the 10 ng/mL-treated group than in the control (Figure 5A; *p* < 0.05) and peaked with 100 ng/mL chemerin treatment (Figure 5A; *p* < 0.05). The increase in the levels of LDH released from H9c2 cardiomyoblasts treated with 100 ng/mL became significant after 12 h, and peaked at 24 h (Figure 5B; both *p* < 0.05). Based on these results, H9c2 cells were treated with 100 mg/mL chemerin for 24 h for subsequent experiments.

### Chemerin-Induced NLRP3 Inflammasome Activation Was Mediated by CMKLR1 and NLRP3

**FIGURE 6:**
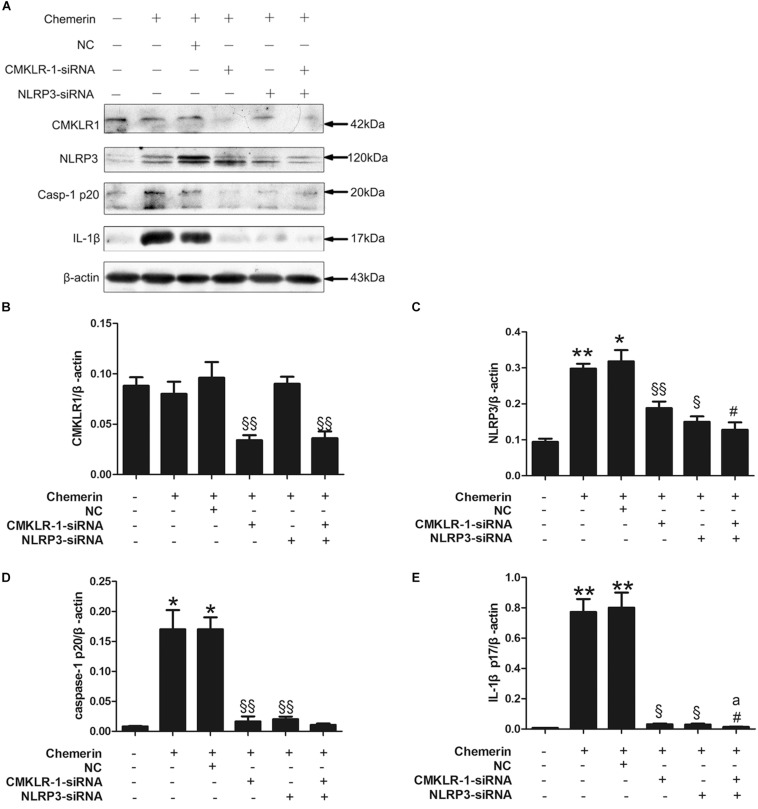
CMKLR1 and NLRP3 induced caspase-1 and IL-1β activation. Relative protein levels of CMKLR1 **(A,B)**, NLRP3 **(A,C)**, activated caspase-1 **(A,D)**, and mature IL-1β **(A,E)** in treated H9c2 cells. Data were presented as means ± SEM from three independent experiments. *p < 0.05, **p < 0.01 vs. Ctrl; ^§^ *p* < 0.05, ^§§^ *p* < 0.01 vs. Chemerin+NC; ^#^*p* < 0.05 vs. Chemerin+CMKLR1-siRNA; ^a^*p* < 0.05 vs. Chemerin+NLRP3-siRNA; NC, negative control.

Chemerin treatment led to increased expression of mature caspase-1 and IL-1β, both of which are markers of NLRP3 inflammasome activation. Compared with the NC, CMKLR1-siRNA treatment decreased the protein levels of CMKLR1 in chemerin-treated H9C2 cells (Figures 6A,B; *p* < 0.01). In addition, CMKLR1-siRNA attenuated the chemerin-induced expression of NLRP3, caspase-1, and IL-1β (Figures 6A,C–E; *p* < 0.05∼*p* < 0.01). NLRP3-siRNA treatment suppressed the chemerin-induced protein levels of activated caspase-1 and IL-1β when compared with the NC (Figures 6A,C–E; *p* < 0.05∼*p* < 0.01). However, NLRP3-siRNA treatment had no effect on the protein level of CMKLR1 (Figures 6A,B). Treatment with siRNAs targeting both CMKLR1 and NLRP3 further decreased the protein levels of activated IL-1β compared with treatment with either siRNA alone (Figures 6A,C–E; *p* < 0.05∼*p* < 0.01).

### NLRP3 Was Involved in Chemerin/CMKLR1-Induced Pyroptosis

**FIGURE 7:**
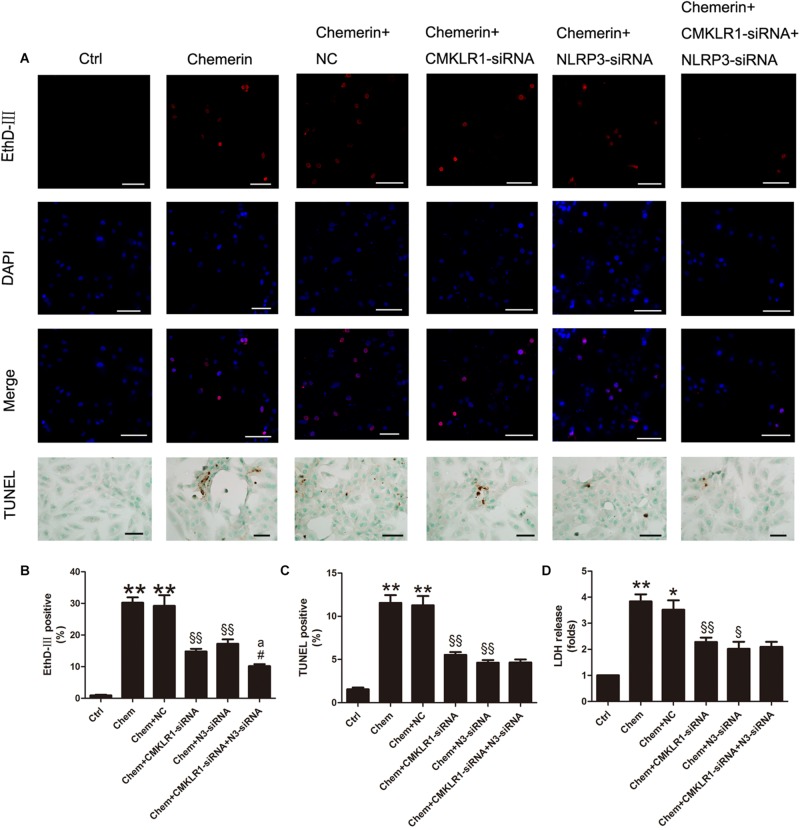
Chemerin/CMKLR1-induced pyroptosis was mediated by NLRP3. **(A)** EthD-III staining (red), DAPI (nuclei; blue), and merged (pink); scale bar: 50 μm. TUNEL staining of H9c2 cardiomyocytes, dark brown staining represent nuclear DNA fragmentation; scale bar: 20 μm. **(B)** Quantitative analysis of EthD-III-positive cells. **(C)** Quantitative analysis of TUNEL-positive cells in each group. **(D)** Relative secreted levels of LDH in the different groups. Data were presented as means ± SEM from three independent experiments. * *p* < 0.05 vs. Ctrl; ** *p* < 0.01 vs. Ctrl; ^§^ *p* < 0.05 vs. DCM+NC; ^§§^ *p* < 0.01 vs. Chemerin+NC; ^#^*p* < 0.05 vs. Chemerin+CMKLR1-siRNA; ^a^*p* < 0.05 vs. Chemerin+NLRP3-siRNA; NC, negative control; Chem, chemerine; N3-siRNA, NLRP3-siRNA.

Results of the EthD-III staining and the LDH release assay showed that cell death and the number of cells with damaged membranes were increased in the chemerin-treated group compared with the control group (Figures 7A,B,D; both *p* < 0.01). Moreover, TUNEL staining results indicated that the levels of cell death associated with nuclear DNA damage were higher in the chemerin-treated group than in the control group (Figures 7A,C; *p* < 0.01). To explore the function of NLRP3 in chemerin/CMKLR1-induced H9c2 cell death, we knocked down the expression of CMKLR1, NLRP3, and CMKLR1+NLRP3 by transfection with the respective siRNAs. Following CMKLR1 knockdown, the ratio of dead cells, as detected by EthD-III, TUNEL, and LDH assays, was lower among CMKLR1-siRNA-treated cells than among NCs (Figures 7A–D; all *p* < 0.01). A similar result was observed in the NLRP3-siRNA-treated group (Figures 7A–D; *p* < 0.05∼*p* < 0.01). Treatment with siRNAs targeting both CMKLR1 and NLRP3 further decreased the ratio of dead cells compared with treatment with either siRNA alone, as determined by EthD-III staining (Figures 7A,B; both *p* < 0.05).

## Discussion

In this study, we found that chemerin could induce inflammation and cardiomyocyte pyroptosis through its receptor, CMKLR1, and these effects were associated with NLRP3-mediated IL-1β and caspase-1 activation. These results indicated that the chemerin/CMKLR1 axis could promote DCM progression and may represent a new therapeutic target for DCM treatment.

In this study, we successfully generated a rat model of DCM that showed the characteristics of cardiac remodeling, cell death, and cardiac dysfunction. The serum chemerin level has been shown to be an important link between inflammation and diabetes-related complications (Zhang et al., 2018; Shang et al., 2019). Consistent with these observations, we found that chemerin expression was upregulated in the serum and left ventricle myocardium of DCM rats. CMKLR1, a well-characterized membrane receptor for chemerin, was also highly expressed in DCM rats and its expression overlapped with that of chemerin. To explore the role of the chemerin/CMKLR1 axis in DCM, we used CMLR1-siRNA *in vivo* to investigate whether inhibition of CMKLR1 could alleviate DCM progression. Because previous studies have shown that cardiac dysfunction in diabetic rats occurs 8 weeks after STZ injection (Lacombe et al., 2007; Ti et al., 2011), we performed CMKLR1-siRNA treatment *in vivo* 8 weeks after STZ injection to explore whether inhibition of CMKLR1 exerted a therapeutic effect on DCM. Four weeks after CMKLR1-siRNA injection, analysis of transfection effectiveness showed that the lentiviral CMKLR1-siRNA vector had worked. No notable adverse effects were observed in lentiviral siRNA-treated rats. CMKLR1 and NLRP3 protein expression was significantly suppressed by CMKLR1-siRNA treatment. Moreover, the expression levels of mature caspase-1 and IL-1β, critical regulators of pyroptosis and inflammation (Lacey et al., 2018; Kelley et al., 2019), were also significantly decreased in CMKLR1-siRNA-treated diabetic rats.

Downregulation of CMKLR1 suppressed cardiac cell death in DCM rats. Although apoptosis and necrosis are the forms of cell death that are normally detected in DCM patients (Chowdhry et al., 2007; D’Arcy, 2019; Ge et al., 2019; Qi et al., 2020) recent studie have shown that other types of cell death, such as pyroptosis, are also found in DCM (Wang X. et al., 2019). Pyroptosis is a type of programmed cell death that is commonly induced by caspase-1 activation, and is characterized by pore formation on the plasma membrane, cell swelling, nuclear DNA damage, and release of intracellular inflammatory content (Liu et al., 2016; Lacey et al., 2018). Notably, we observed the characteristic hallmarks of pyroptosis in the left ventricles of DCM rats, including cytoplasmic swelling, nuclear DNA damage, and increased activation of caspase-1. This result suggested that pyroptosis had occurred during DCM progression.

*In vitro*, DCM is often imitated in rat embryonic heart derived H9C2 cells, neonatal cardiomyocytes or cardiac fibroblasts with high glucose or other kind of cytokine stimulation. H9c2 cells were used in our study to explore the mechanism of chemerin-regulated cell death. Chemerin induced the release of LDH from H9c2 cells, indicating that the cell membrane was disrupted. NLRP3 inflammasome activation led to caspase-1 activation, along with pore formation in the plasma membrane and nuclear DNA damage in chemerin-treated H9c2 cells. These data supported our hypothesis that pyroptosis occurred in cardiomyocytes. The TUNEL result indicated that apoptosis also contributed to the DCM-induced cell death. Knockdown of CMKLR1 *in vivo* and *in vitro* markedly inhibited caspase-1 activation, which was accompanied by decreased pyroptosis. These results indicated that caspase-1-dependent pyroptosis is a contributing factor to chemerin-induced cell death in DCM. The chemerin/CMKLR1 axis was shown to be a critical regulator of pyroptosis in DCM. The role of the chemerin/CMKLR1 axis in apoptosis requires further investigation.

The DCM rats showed myocardial lipid accumulation, which can result in cardiac lipotoxicity (Li et al., 2019) and was thus likely to have contributed to DCM. After CMKLR1 downregulation, the cardiac lipid imbalance was markedly reversed.

DCM rats showed substantial hypertrophy and fibrosis in the myocardium as determined by H&E and Masson’s staining. Cardiac hypertrophy and fibrosis, common pathological changes in DCM, can induce left ventricular stiffness (Wang Y. et al., 2019; Yuan et al., 2019; Luo et al., 2020). Consistent with this, diastolic and systolic dysfunction were observed by echocardiographic analysis in DCM rats. Knockdown of CMKLR1 alleviated the aberrant hypertrophy and fibrosis in DCM. Furthermore, both the systolic and diastolic dysfunction of the left ventricle were improved in CMKLR1-siRNA-treated DCM rats, as evidenced by the attenuated hypertrophy, reduced fibrosis, decreased cardiac pyroptosis, and reduced myocardial lipid deposition. These observations showed that CMKLR1-siRNA treatment exerts a therapeutic effect on DCM.

The chemerin/CMKLR1 axis has been proposed to regulate the activation of the NLRP3 inflammasome in Kupffer cells in a mouse model of nonalcoholic fatty liver disease (Zhang et al., 2017), and a rat model of limb ischemia/reperfusion-acute lung injury (Zou et al., 2019). In line with these findings, our study showed that chemerin promoted caspase-1 and IL-1β activation in H9c2 cardiomyocytes, which could be markedly suppressed by treatment with CMKLR1-siRNA. The results indicated that chemerin induced NLRP3 inflammasome activation through the CMKLR1 receptor. Chemerin-induced mature caspase-1 and IL-1β overexpression was suppressed following NLRP3-siRNA treatment, indicating that NLRP3 is an important mediator of chemerin/CMKLR1-regulated inflammasome activation. Interestingly, treatment with siRNAs targeting both CMKLR1 and NLRP3 suppressed the expression of mature IL-1β, but not that of caspase-1, to a greater extent than treatment with either siRNA alone. These results indicated that CMKLR1 is an important, although not exclusive, mediator of chemerin-induced NLRP3 inflammasome activation, and that the chemerin/CMKLR1 axis may regulate caspase-1 and IL-1β activation primarily through NLRP3. The EthD-III staining, LDH release assay, and TUNEL staining results indicated that both CMKLR1-siRNA and NLRP3-siRNA treatments could attenuate cell membrane and nuclear DNA damage. Interestingly, EthD-III staining also revealed that the rate of pyroptosis with CMKLR1+NLRP3 knockdown was decreased to a greater extent than with knockdown of either CMKLR1 or NLRP3 alone. These results indicated that NLRP3 inflammasome-regulated caspase-1 was a critical but not exclusive link between the chemerin/CMKLR1 axis and pyroptosis.

In conclusion, our results revealed that the chemerin/CMKLR1 axis played an important role in the inflammation, hypertrophy, pyroptosis, and fibrosis that occurs in the heart tissue of DCM rats. The effect of the chemerin/CMKLR1 axis on inflammation and pyroptosis was mainly mediated by the NLRP3 inflammasome. Caspase-1-regulated pyroptosis played an important role in the pathogenesis of DCM. Gene interference targeting *CMKLR1* seems to be effective for treating DCM in a rat model. Further studies are needed to validate the targeting of chemerin/CMKLR1 as a potential therapeutic strategy for the treatment of DCM.

## Data Availability Statement

The raw data supporting the conclusions of this manuscript will be made available by the authors, without undue reservation, to any qualified researcher.

## Ethics Statement

The study protocol was approved by the Institutional Animal Care and Use Committee of the Guangxi Medical University.

## Author Contributions

BL and YX were responsible to induce animal model and cell experiment. YH performed immunohistochemistry staining and other staining. XL analyzed and interpreted the animal model data. HQ performed the ultrasonic cardiogram examination of the heart. MW carried out western blot experiment. BL conceived the manuscript. All authors read and approved the final manuscript.

## Conflict of Interest

The authors declare that the research was conducted in the absence of any commercial or financial relationships that could be construed as a potential conflict of interest.
